# Glabridin improves autoimmune disease in Trex1-deficient mice by reducing type I interferon production

**DOI:** 10.1186/s10020-023-00754-y

**Published:** 2023-12-08

**Authors:** Jincai Wen, Wenqing Mu, Hui Li, Yulu Yan, Xiaoyan Zhan, Wei Luo, Zhongxia Wang, Wen Kan, Jia Zhao, Siwen Hui, Ping He, Shuanglin Qin, Yingjie Xu, Ping Zhang, Xiaohe Xiao, Guang Xu, Zhaofang Bai

**Affiliations:** 1 Department of Hepatology, The Fifth Medical Center of Chinese, PLA General Hospital, Beijing, 100039 China; 2 State Key Laboratory of Radiation Medicine and Protection, Institutes for Translational Medicine, Soochow University, Suzhou, 215123 Jiangsu China; 3 Ningde Hospital of Traditional Chinese Medicine, Fujian University of Traditional Chinese Medicine, Fuzhou, China; 4 Fifth Medical Center of Chinese, China Military Institute of Chinese Materia, PLA General Hospital, Beijing, 100039 China; 5 Nutrition Department of the Fifth Medical Center of the PLA General Hospital, Beijing, 100039 China; 6 School of Pharmacy, Xianning Medical College, Hubei University of Science and Technology, Xianning, People’s Republic of China; 7 Department of Pharmacy, Medical Supplies Center of PLA General Hospital, Beijing, 100039 China; 8 School of Traditional Chinese Medicine, Capital Medical University, Beijing, 100069 China; 9 National Key Laboratory of Kidney Diseases, Beijing, 100005 China

**Keywords:** Glabridin, cGAS-STING, IRF3, Autoimmune diseases, Type I interferon

## Abstract

**Background:**

The cGAS-STING signaling pathway is an essential section of the natural immune system. In recent years, an increasing number of studies have shown a strong link between abnormal activation of the cGAS-STING signaling pathway, a natural immune pathway mediated by the nucleic acid receptor cGAS, and the development and progression of autoimmune diseases. Therefore, it is important to identify an effective compound to specifically downregulate this pathway for disease.

**Methods:**

The effect of Glabridin (Glab) was investigated in BMDMs and Peripheral blood mononuclear cell (PBMC) by establishing an in vitro model of cGAS-STING signaling pathway activation. An activation model stimulated by DMXAA was also established in mice to study the effect of Glab. On the other hand, we investigated the possible mechanism of action of Glab and the effect of Glab on Trex1-deficient mice.

**Results:**

In this research, we report that Glab, a major component of licorice, specifically inhibits the cGAS-STING signaling pathway by inhibiting the level of type I interferon and inflammatory cytokines (IL-6 and TNF-α). In addition, Glab has a therapeutic effect on innate immune diseases caused by abnormal cytoplasmic DNA in Trex1-deficient mice. Mechanistically, Glab can specifically inhibit the interaction of STING with IRF3.

**Conclusion:**

Glab is a specific inhibitor of the cGAS-STING signaling pathway and may be used in the clinical therapy of cGAS-STING pathway-mediated autoimmune diseases.

## Introduction

Natural immunity is the innate defense ability of the organism. Natural immunity is triggered primarily by damage-associated molecular patterns, which recognize pathogen-associated molecular patterns and pattern-recognition receptors that immunize the body against foreign pathogens, and is the first line of defense of natural immunity to protect the organism (Liu et al. 2022; Lockhart et al. 2022). The cGAS-STING signaling pathway is an important component of the natural immune system that has been discovered in recent years (Kwon and Bakhoum 2020; Zhao et al. 2023). The cGAS is a novel cytoplasmic DNA receptor that recognizes abnormal DNA present in the cytoplasm produced by the organism or from outside and catalyzes the formation of cyclic GMP-AMP (cGAMP) from ATP and GTP, which binds as a second messenger and activates STING located in the endoplasmic reticulum (Zhang et al. 2020). Binding of GAMP and STIING results in translocation of STING from the endoplasmic reticulum to the Golgi apparatus, eventually transferred to the vesicles in the perinuclear region (Wang et al. 2020). Meanwhile, STING can recruit TANK-binding kinase 1 (TBK1) to form a complex that catalyzes phosphorylation and dimerization of IRF3, which is able to translocate from the cytoplasm to the nucleus and induce transcription of innate immune genes, ultimately leading to increased expression of type I interferon and inflammatory factors (Long et al. 2022; Zhuang et al. 2022).

Although the cGAS-STING signaling pathway plays a very important role in the treatment of innate immune and inflammatory diseases, inappropriate activation of this pathway can also trigger autoimmune and inflammatory diseases such as familial frostbite lupus erythematosus, neuroinflammatory and neurodegenerative diseases, and Parkinson's disease (Zheng et al. 2021). Available studies have shown that the cGAS-STING pathway triggers an autoinflammatory response in three-prime repair exonuclease 1 *(Trex1*) -deficient mice, with lethality occurring at around week eight (Maltbaek et al. 2022). *Trex1* is a very important endogenous DNA nucleic acid exonuclease in cells that can remove DNA fragments that need to be processed by hydrolyzing damaged DNA in damaged cells, thus avoiding immune hyperactivation and autoimmune diseases (Mathavarajah et al. 2019; Nader et al. 2021). It has also been shown that if IRF3 is simultaneously knocked out in Trex1 mice, autoantibody and type I interferon production can be effectively reduced, thereby avoiding death, which also suggests that autoimmune diseases in *Trex1*^*−/−*^ mice are associated with IRF3-dependent type I interferon production (Stetson et al. 2008). In addition, for inflammatory diseases caused by the cGAS-STING pathway, small molecule compounds have been reported as antagonists of the cGAS-STING pathway, such as RU.521, C-176 and H151 (Zhou et al. 2018; Hopfner and Hornung 2020; Wang et al. 2022). Furthermore, DMXAA and CMA, two synthetic small molecule compounds that promote STIING-dependent signalling in mice, have the disadvantage of not activating human STING (Graham et al. 2022). Therefore, it is of significance to explore the development of safe and effective inhibitors of the cGAS-STING pathway for the treatment of cGAS-STING-mediated inflammatory diseases.

Glabridin (Glab) is a flavonoid component unique in the Chinese herb licorice, one of the ancient and commonly used herbs (Chung et al. 2022; Ge et al. 2023). It has been shown that licorice flavonoid components have good anti-inflammatory effects and are protective against both liver injury and lung injury (Li et al. 2022; Wen et al. 2023), while Glabridin can significantly inhibit the free radicals generated during human metabolism and prevent some pathological diseases related to free radical oxidation (Yehuda et al. 2016). It also has the ability to improve cardiovascular disease, inhibit melanin formation and have anti-inflammatory effects. The apparent beneficial effects of Glabridin are related to its structure, but its potential mechanism of action and immediate targets are still not well understood and no inhibitors of the cGAS-STING signalling pathway have yet been approved (Dai et al. 2021; Zhang et al. 2022a, b).

In the present study, we found that Glabridin specifically inhibited the activation of the cGAS-STING signaling pathway and that mice given Glabridin attenuated the increase in interferon and inflammatory factors induced by DMXAA. Mechanistically, Glabridin inhibits the interaction of STING with IRF3. Collectively, this study shows that Glabridin is a small molecule inhibitor of the cGAS-STING pathway and is expected to provide a reference for the development of inhibitors of the cGAS-STING pathway.

## Materials and methods

### Animals

C57BL/6 J female mice, weighing 18–20 g, aged 8 weeks, were obtained from SPF Biotechnology Co., Ltd. (Beijing, China). Trex1^−/−^ mice were licensed by Dr. Tao Li from the National Center of Biomedical Analysis (NCBA) (Beijing, China), male and female *Trex1*^−/−^ mice were further mated to generate mice that were genotyped by standard PCR, and 4-week-old littermates of Trex1^−/−^ mice were started with injections of excipients and Glab. The animals are kept in a suitable environment with free access to food and water, and bedding is changed on time. All animal manipulations in the experiments were performed in accordance with the regulations and with the least possible suffering of the mice and the number of mice used in the experiments.

### Cell culture

The Bone-marrow-derived macrophages (BMDMs) used in this study were obtained from healthy female mice, then cultured in Dulbecco’s modified Eagle’s medium (DMEM) containing 10% Certified  fetal bovine serum (FBS, VivaCell, Shanghai, China), 1% penicillin/streptomycin and 50 ng/mL murine macrophage colony-stimulating factor (MCSF). PBMC is obtained from the blood of healthy volunteers and later cultured in RPMI-1640 medium. HEK-293 and HEK-293 T cells were cultured in DMEM. All cells were grown according to the requirements in the ATCC database.

### Antibodies and reagents

**Table:** 

Antibodies and Reagents		
Rabbit monoclonal anti-Phospho-IRF-3	GeneTex	GTX86691
Rabbit monoclonal anti-Phospho-IRF-3	Abcam	ab76493 Ser386
IRF3 polyclonal antibody	Proteintech	11312–1-AP
TMEM173/STING polyclonal antibody	Proteintech	19851–1-AP
TBK1 polyclonal antibody	Proteintech	28397–1-AP
HSP90 polyclonal antibody	Proteintech	13171–1-AP
Glabridin	MedChemExpress	HY-N0393
2′3'-cGAMP	APEBIO	B8362
Herring testis DNA	Sigma	D6898
DMSO	Sigma	D2650
Protease inhibitor cocktail	C0001	TargetMol
Mouse IFN-β ELISA kit	Invivogen	luex-mifnbv2
Mouse IL-6 ELISA kit	Dakewe	1210602
Mouse TNF-α ELISA kit	Dakewe	1217202
StarFect Transfection Reagent	GenStar	C101-10
RT Master Mix for qPCR II	MedChemExpress	HY-K0510A
SYBR Green qPCR Master Mix (Low ROX) Anti-FLAG® M2 Affinity Gel Certified Fetal Bovine Serum	MedChemExpress Sigma VivaCell,	HY-K0522 A2220 C04001

### Activation of the cGAS-STING signaling pathway

In vivo experiments, we selected healthy 8-week-old female C57BL/6 J mice. Mice were injected i.p. with Glab (20 or 40 mg/kg) for 1 h and were then injected i.p. with DMXAA (25 mg/kg). Four hours later, we sacrificed the mice, collected the serum samples, and washed the mice’s abdominal cavity with pre-cooled PBS to obtain the peritoneal lavage solution.

In the Trex1^−/−^ mice autoinflammatory response assay, WT and Trex1^−/*−*^ mice were housed together and Glab (40 mg/kg) was injected intraperitoneally daily for 14 days, then the heart, tongue, muscle, stomach, kidney and intestine tissues were collected, part of this is used to detect mRNA expression of interferon-related genes and inflammatory factors by qPCR, and another portion is used for histological examination.

In vitro experiments, we inoculated BMDMs at 1 × 10^6^ cells/mL with 1.0 mL in 12-well plates, and after the cells were fully apposed, BMDMs were treated with DMSO or Glab for 1 h, then stimulated with HT-DNA and Poly(I:C) for 2 h. Cells were lysed by loading with 1 × RIPA and whole cell lysates were collected, and then Western Blots analysis of the corresponding protein expression was performed.

### Western blotting

The specific experimental steps and precautions for western blotting are described previously (Wang et al. 2019).

### Enzyme-linked immunosorbent assay

The secretion of IFN-β in serum and peritoneal lavage fluid samples was measured by Mouse IFN-β ELISA kit (Invivogen, luex-mifnbv2). IL-6 and TNF-α secretion measured by Mouse IL-6 ELISA kit (1210602, Dakewe Beijing) and Mouse TNF-α ELISA kit (1217202, Dakewe, Beijing). All experimental steps were performed according to the instructions.

### STING oligomerization assay

The STING oligomerization assay was performed as described previously (Li et al. 2018a, b). BMDMs were first treated with DMSO and Glab for 1 h and then stimulated with cGAMP for 2 h. Cells are lysed with a lysis solution [50 mM Tris–HCl, 150 mM NaCl, 0.5% Triton X-100, (pH 7.5)] containing the protease inhibitor at 4 °C for 15 min, with shaking at intervals of several minutes to allow fuller lysis. After centrifugation at 4 °C, 8000 × g for 15 min, the cell supernatant was collected. Then adding 1 × loading without SDS, it was subsequently loaded into a natural PAGE gel without SDS and run at 80 mA for 2 h, followed by immunoblotting with the corresponding antibody.

### Transfection experiments

HEK-293 cells were planted at a density of 5 × 10^5^ in 24-well plates at 0.5 mL per well. after complete cells were fully apposed. the cells were transfected with Flag-tagged plasmids (STING, IRF3, TBK1, MAVS) for 12 h and then treated with 20 μM Glab for 6 h. after 6 h, samples for WB were collected by lysis with 1 × RIPA loading and samples for qPCR were lysed with Trizol.

### Immunoprecipitation

HEK-293 T cells were seeded at a density of 5.5 × 10^5^ in 6-well plates at 2 mL per well., and after complete apposition, cells were transfected with Flag-tagged plasmids (Flag-Vector, Flag-IRF3) and HA-tagged plasmids (HA-Vector, HA-STING) for 18 h. Subsequently, treated with 20 μM Glab for 6 h. Cells were lysed with the lysis buffer (50 mM Tris–HCl [pH 7.5], 150 mM NaCl, 0.25% Sodium deoxycholate, 1% Triton X-100, 0.5% NP-40, 2 mM EDTA) containing complete protease inhibitor cocktail. The lysate was collected and centrifuged at 4 °C, 12000 × g for 15 min, then a portion of the supernatant was aspirated as input. For immunoprecipitation, the supernatant was incubated with Anti-FLAG® M2 Affinity Gel for 4 h according to the instructions. Followed by centrifugation at 4 °C, 750 × g for 5 min, flipped to mix well, and washed with lysate, and the above operation was repeated 4 times. The samples were then added to 1 × SDS-PAGE loaded immunoprecipitation analysis buffer, followed by immunoblotting with the appropriate antibodies to analyses the expression of the corresponding proteins.

### cDNA preparation and quantitative PCR

We seeded BMDMs at 1 × 10^6^ cells/mL with 1.0 mL in 12-well plates. After the cells were fully apposed, they were treated with DMSO or Glab of various concentrations for 1 h, followed by different stimuli for 4 h. Discard the cell supernatant, lyse the cells with Trizol, and then collect in 1.5 mL EP tubes and store at − 20 °C. For RNA extraction, Follow the RNA extraction kit to perform the RNA extraction operation. After reverse transcription with RT Master Mix for qPCR II (MedChemExpress, HY-K0510A), qRT-PCR was performed with SYBR Green qPCR Master Mix (Low ROX) (MedChemExpress, HY-K0522).

### Statistical analyses

In this study, we used GraphPad 8.0 software and Excel to perform statistical analysis of the sample values. All experimental data are expressed as mean ± standard deviations (SEM). The unpaired student *t*-test was used to compare the statistical differences between the two groups. One-way ANOVA with Dunnett’s post hoc test was used to compare statistical differences between multiple groups. The difference was considered statistically significant at ^*^P < 0.05, ^**^P < 0.01, and ^***^P < 0.001. NS, not significant.

### Quantitative PCR primer sequences

RT-PCR gene selection according to MIQE guidelines (Bustin et al. 2009).

**Table:** 

Target gene name	Gene Sequence (5ʹ–3ʹ)	Source
Human Actin	CATGTACGTTGCTATCCAGGC	From PMID: 33142842
	CTCCTTAATGTCACGCACGAT	
Human IFN-β	TCCAAATTGCTCTCCTGTTG	From PMID: 35577759
	GCAGTATTCAAGCCTCCCAT	
Human IL-6	ACTCACCTCTTCAGAACGAATTG	From PMID: 27286733
	CCATCTTTGGAAGGTTCAGGTTG	
Human TNF-α	CCTCTCTCTAATCAGCCCTCTG	From PMID: 27286733
	GAGGACCTGGGAGTAGATGAG	
Mouse Actin	GGCTGTATTCCCCTCCATCG	From prime bank,
	CCAGTTGGTAACAATGCCATGT	ID: 11461
Mouse IFN-β	TCCGAGCAGAGATCTTCAGGAA	From PMID: 35577759
	TGCAACCACCACTCATTCTGAG	
Mouse IL-6	CACTTCACAAGTCGGAGGCT	From PMID: 27286733
	CTGCAAGTGCATCATCGTTGT	
Mouse TNF-α	GGGCAGTTAGGCATGGGAT	From PMID: 27286733
	TGAGCCTTTTAGGCTTCCCAG	
Mouse CXCL10	ATCATCCCTGCGAGCCTATCCT	From PMID: 27286733
	GACCTTTTTTGGCTAAACGCTTTC	
Mouse ISG15	GGTGTCCGTGACTAACTCCAT	From prime bank,
	CTGTACCACTAGCATCACTGTG	ID 226874850c1

## Results

### Glab inhibits the activation of cGAS-STING signaling pathway in BMDMs

**Fig. 1:**
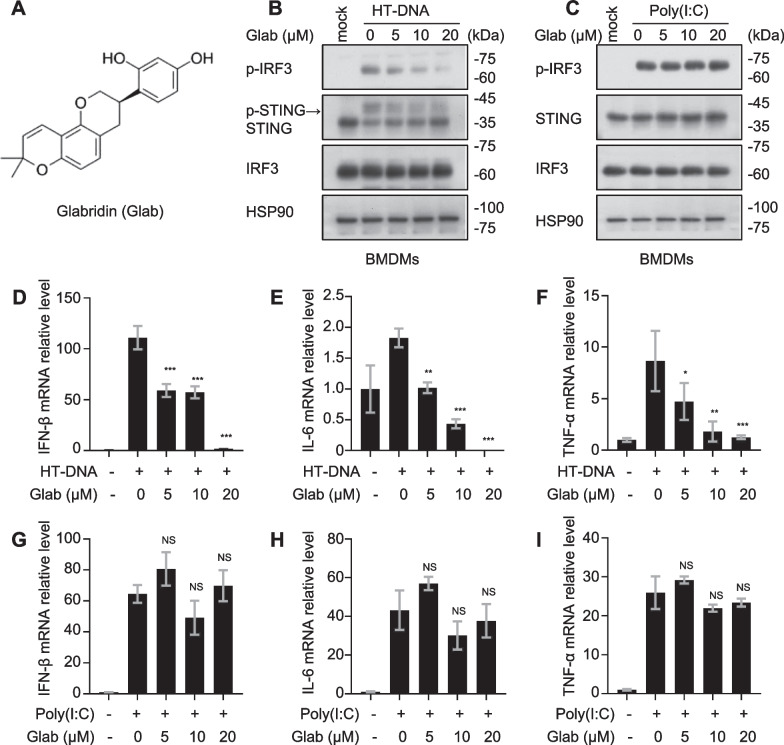
Glab inhibits the activation of the cGAS-STING signaling pathway. **A** Glabridin (Glab) structure. **B** and **C** BMDMs were first treated with DMSO or Glab of various concentrations for 1 h, and then stimulated with HT-DNA or Poly(I:C) for 2 h. Whole cell lysate (WCL) was collected and immunoblotted with the indicated antibody. **D**–**F** BMDMs were treated with DMSO or Glab of various concentrations for 1 h, and then stimulated with HT-DNA for 4 h. The expression of IFN-β, IL-6 and TNF-α mRNA level were detected by quantitative polymerase chain reaction (qPCR) assay. **G**–**I** BMDMs were treated with DMSO or Glab of various concentrations for 1 h and then stimulated with Poly(I:C) for 4 h. The expression of IFN-β, IL-6 and TNF-α mRNA were detected by qPCR assay. Data in **D**–**I** are presented as mean ± SEM from three biological replicates. one-way ANOVA and Dunnett’s post hoc test were used to assess the differences of multiple groups, ^*^p < 0.05, ^**^p < 0.01 and ^***^p < 0.001 vs. the control, NS, not significant

To assess the effect of Glab (Fig. 1A) in licorice on the cGAS-STING signaling pathway. In BMDMs, the cGAS-STING signaling pathway was first treated with Glab with a certain concentration for 1 h and then activated with herring testes DNA (HT-DNA), an abnormal double-stranded DNA that, upon entering the cytoplasm, is sensed by the signaling receptor cGAS, which activates the cGAS-STING pathway. As shown in Fig. 1B, Glab treatment dramatically inhibited the activation of STING and IRF3 in BMDMs. In parallel, we examined the gene expression of downstream type I interferons and pro-inflammatory factors activated by HT-DNA after Glab treatment. As expected, the expression of IFN-β, IL-6 and TNF-α were reduced after Glab treatment (Fig. 1D–F). In addition, poly(I:C) is a synthetic aberrant RNA that activates the Rig-1-MAVS pathway, which can also lead to increased IRF3 activation and expression of interferon-related genes as well as inflammatory factors. However, in our experiments, Glab did not affect the expression of related proteins and genes under poly(I:C) stimulation (Fig. 1C, G–I). In summary, these data suggest that Glab can specifically inhibit the activation of cGAS-STING signaling pathway in BMDMs.

### In BMDMs and human PBMCs, Glab is a broad-spectrum inhibitor of the cGAS-STING pathway

**Fig. 2:**
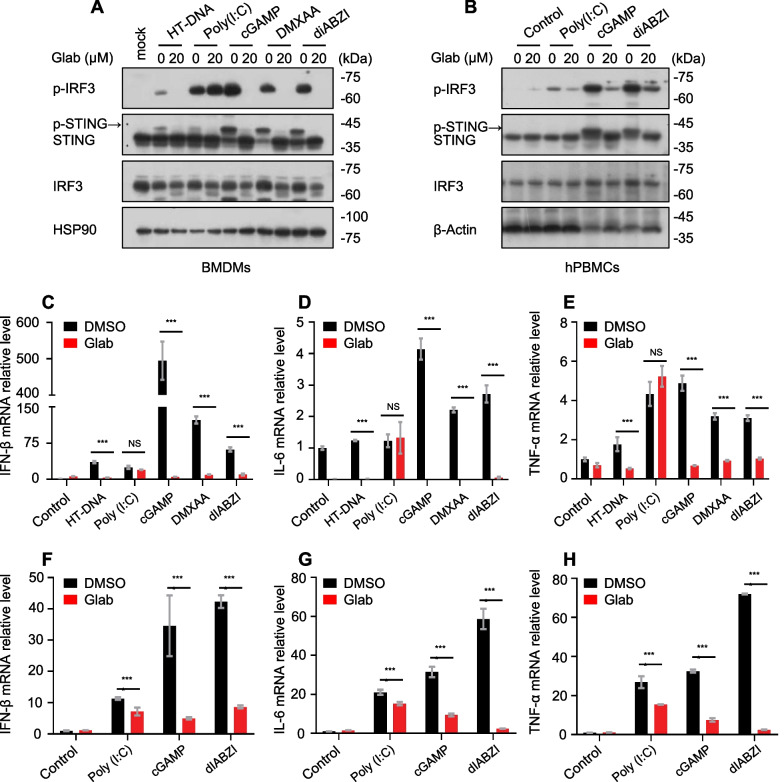
Glab is a specific inhibitor of the cGAS-STING signaling pathway. **A** BMDMs were first treated with DMSO or Glab (20 μM) for 1 h, then stimulated with HT-DNA, Poly(I:C), cGAMP, DMXAA and diABZI to analyze the phosphorylation of IRF3 and the expression of STING in whole cell lysates (WCL) by immunoblotting. **B** Human PBMCs were first treated with DMSO or Glab (20 μM) for 1 h, and then stimulated with Poly(I:C), cGAMP and diABZI for 2 h. Whole cell lysate was collected and immunoblotted with the indicated antibody. (**C–E**) BMDMs were first treated with Glab (20 μM) for 1 h and then stimulated with HT-DNA, Poly(I:C), cGAMP, DMXAA and diABZI for 4 h. The expression of IFN-β, IL-6 and TNF-α mRNA were detected by qPCR assay. **F**–**H** Human PBMCs were first treated with Glab (20 μM) for 1 h and then stimulated with HT-DNA, Poly(I:C), cGAMP and diABZI for 4 h. The expression of IFN-β, IL-6 and TNF-α mRNA were detected by qPCR assay. Data in (**C–H**) are expressed as mean ± SEM (n = 3/group, from three biological replicates.). Unpaired student *t*-test ** C–H** was used to assess differences among groups, ^*^p < 0.05, ^**^p < 0.01 and ^***^p < 0.001 vs. the control, NS, not significant

To verify whether Glab has a broad-spectrum inhibitory effect on the cGAS-STING signaling pathway, we observed the effect of Glab on the cGAS-STING pathway induced by various stimuli. cGAMP, DMXAA and dIABZI are all agonists that target STING and all activate the cGAS-STING signalling pathway at the cellular level. During the study, it was found that Glab effectively inhibited STING and IRF3 activation induced by these agonists in BMDMs (Fig. 2A). Also, at the mRNA level we obtained the same results (Fig. 2C–E). On the other hand, the same experiments were performed in human peripheral blood mononuclear cells (PBMCs) obtained from healthy volunteers. We found that Glab treatment effectively inhibited the activated IRF3 (where DMXAA can only activate the cGAS-STING signalling pathway in mouse cells) (Fig. 2B), the expected results were obtained by detecting the gene expression of both type I interferon and pro-inflammatory factors (Fig. 2F–H). Notably, in PBMCs, a certain degree of inhibition of phosphorylated IRF3 appeared in response to Poly(I:C) stimulation, and we speculate that this phenomenon may be due to the fact that the corresponding molecular mechanisms of action are also different in different cells. Taken together, these results suggest that Glab is a broad-spectrum inhibitor of the cGAS-STING signaling pathway in BMDMs and human PBMCs.

### Glab inhibits the activation of STING and downstream signaling pathway in Vivo

**Fig. 3:**
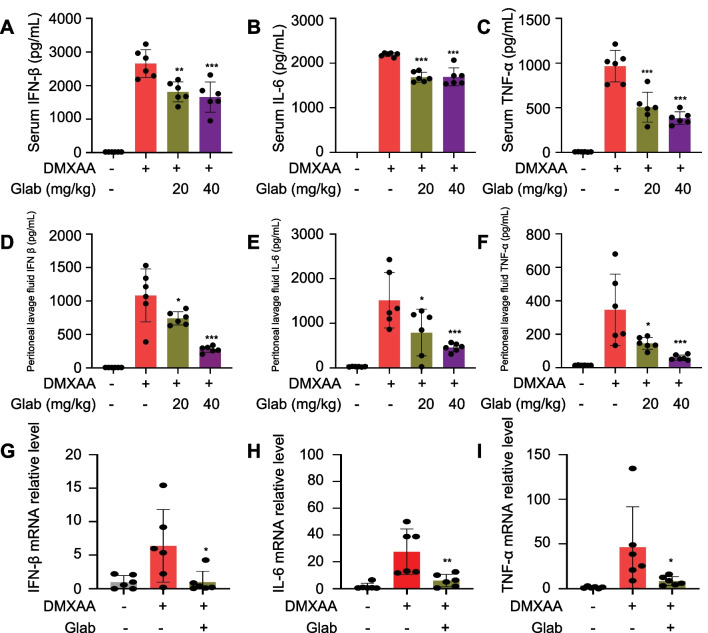
Glab inhibits the activation of STING and downstream signaling pathway in vivo. **A**–**C** Determination of IFN-β (**A**), IL-6 (**B**) and TNF-α (**C**) concentrations in collected serum by ELISA kits (n = 6 mice per group). **D**–**F** Determination of IFN-β (**D**), IL-6 (**E**) and TNF-α (**F**) concentrations in collected peritoneal lavage fluid by ELISA kits (n = 6 mice per group). **G**–**I** Cells were collected by centrifugation of peritoneal lavage fluid and analyzed for mRNA expression of relevant genes by qPCR assay (n = 6 mice per group). Data in (**A–I**) are expressed as mean ± SEM (n = 6 mice per group). one-way ANOVA and Dunnett's post hoc test were used to assess differences among groups, ^*^p < 0.05, ^**^p < 0.01 and ^***^p < 0.001 vs. the control, NS, not significant

Moreover, we investigated the effect of Glab on the cGAS-STING pathway in mice in vivo. DMXAA is a molecule agonist that targets STING and activates the cGAS-STING pathway in mice (Ding et al. 2020). In this experiment, we set up the low-dose (20 mg/kg) and high-dose group (40 mg/kg). One hour after intraperitoneal injection of Glab, mice were injected with DMXAA (25 mg/kg), serum and peritoneal lavage fluid were collected 4 h after stimulation. Under the activation of DMXAA, mice will undergo a severe inflammatory response within a short period of time, and the level of inflammatory factors in the serum will be elevated, as well as the inflammatory response of single nucleated cells and macrophages in the peritoneal cavity of mice. The levels of IFN-β, IL-6 and TNF-α in serum and peritoneal lavage fluid were then measured by ELISA kits. The results showed that the levels of IFN-β, IL-6 and TNF-α were increased in the model group, while the levels of the factors in the serum and peritoneal lavage fluid were decreased after Glab treatment (Fig. 3A–F). We also obtained the same results by analyzing the mRNA levels of IFN-β, IL-6 and TNF-α in the cells in the peritoneal lavage fluid (Fig. 3G–I). Combined with the above results, it shows that Glab can in organisms effectively inhibit the activation of cGAS-STING signaling pathway.

### Glab inhibits the cGAS-STING pathway by disrupting the STING-IRF3 interaction

From the above results, we know that Glab can effectively inhibit the cGAS-STING signaling pathway. It has been shown that by transfecting key node proteins on the signalling pathway (such as MAVs, STING, TBK1 and IRF3), it can also lead to the activation of downstream signalling pathways (Li et al. 2018a, b). To determine the specific target proteins of Glab, Flag-tagged plasmids (MAVS, STING, TBK1 and IRF3) were overexpressed in HEK-293 cells in the presence or absence of Glab, and the expression of IFN-β mRNA level was detected by qPCR assay.

**Fig. 4:**
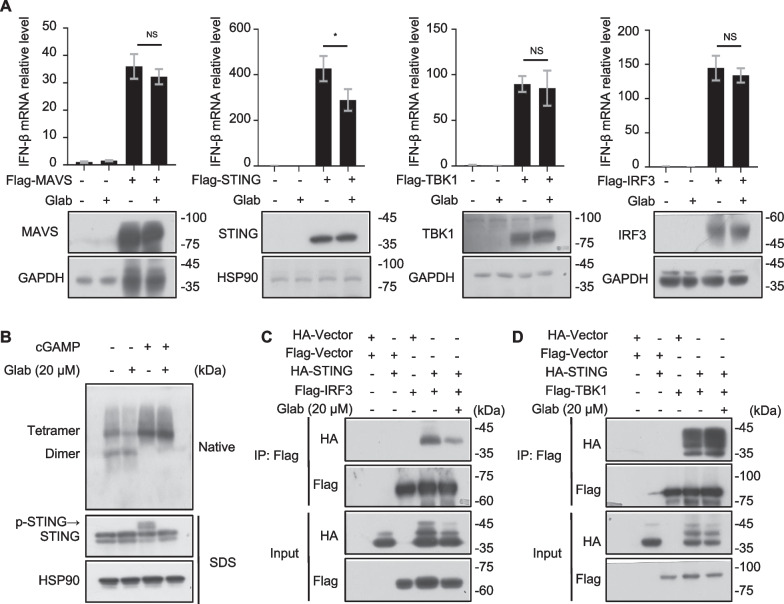
Glab inhibits the activation of the cGAS-STING pathway by disrupting the STING-IRF3 interaction. **A** Transfection of Flag-tagged plasmids (Flag-MAVS, Flag-STING, Flag-TBK1 and Flag-IRF3) into HEK-293 cells for 12 h, and then treated with vehicle, Glab (20 μM) for 6 h. Whole cell lysates were collected and immunoblotted with the indicated antibody. Samples for qPCR were detected by qPCR assay for the expression of IFN-β mRNA. **B** BMDMs were first treated with DMSO or Glab (20 μM) for 1 h and then stimulated with cGAMP for 2 h. The expression of STING and the oligomerization of SITNG in the cell lysate were analyzed by Western blot with the indicated antibodies. **C** and **D** HEK-293 T cells were transfected with Flag-tagged plasmids (Flag-Vector, Flag-IRF3 and Flag-TBK1) and HA-tagged plasmids (HA-Vector and HA-STING) for 20 h, then treated with Glab (20 μM) for 6 h and immunoprecipitated with Anti-FLAG® M2 Affinity Gel, as shown by Western Blots analysis. Data in **A** are expressed as mean ± SEM (n = 3/group, from three biological replicates.). one-way ANOVA and Dunnett’s post hoc test were used to assess differences among groups, ^*^p < 0.05, ^**^p < 0.01 and ^***^p < 0.001 vs. the control, NS, not significant

The results showed that Glab treatment reduced the expression of IFN-β mRNA caused by transfection with Flag-STING and there was no effect on the expression of genes induced by overexpression of MAVS, TBK1 or IRF3 (Fig. 4A). Based on the experimental results, we guessed that Glab might act on STING. Further study we wanted to know if Glab had an effect on the oligomerization of STING and the results showed no effect (Fig. 4B). Furthermore, STING oligomerizes and undergoes translocation upon binding to cGAMP, which then recruits and activates the TBK1 kinase, and the transcription factor IRF3 with STING-TBK1 signalosome is subsequently recruited and activated, which then enters the nucleus to regulate the expression of relevant genes (Zhao et al. 2023). In HEK-293 T cells we investigated the exact mechanism of the Glab action on the cGAS-STING signalling pathway by transfecting the exogenous plasmids (HA-STING, Flag-TBK1 and Flag-IRF3). The results showed that treatment of Glab in HEK-293 T cells inhibited the interaction between STING and IRF3, but had no effect on the binding of STING-TBK1 (Fig. 4C–E). In conclusion, the results suggest that Glab acts to inhibit the cGAS-STING signaling pathway by acting on STING and disrupting the interaction between STING and IRF3.

### Glab alleviates the autoinflammatory response in Trex1^−/*−*^ mice

**Fig. 5:**
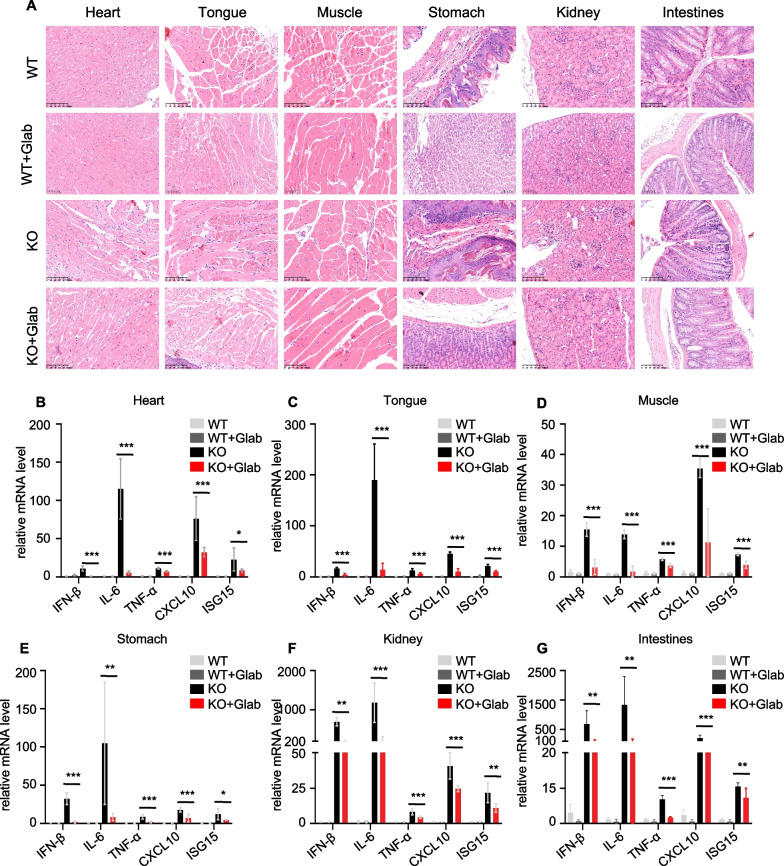
Glab Alleviates the Autoinflammatory Response in Trex1^−/−^ Mice. **A** Representative images of various tissues stained with hematoxylin–eosin (H&E) staining (n = 6 mice per group). **B**–**G** WT mice (n = 6 per group) or Trex1^−/−^ Mice (n = 6 per group) were injected intraperitoneally with 40 mg/kg Glab or vehicle every other day for 14 consecutive days to incorporate the syndrome. Then, heart (**B**), muscle (**C**), tongue (**D**), stomach (**E**), kidney (**F**) and Intestines (**G**) tissues were collected and analyzed the mRNA expression of the indicated genes was determined by qPCR assay. Data in (**B**-**G**) are expressed as mean ± SEM (n = 6 mice per group). one-way ANOVA and Dunnett’s post hoc test were used to assess differences among groups, ^*^p < 0.05, ^**^p < 0.01 and ^***^p < 0.001 vs. the control, NS, not significant

Previous studies have concluded that Glab has a great inhibitory effect on the cGAS-STING signalling pathway in vitro. We speculated whether Glab could mitigate immune hyperactivation and AID. A link between compound targeting of STING and auto-inflammatory responses in *Trex1*^*−/*−^ mice has also been recently demonstrated (Vanpouille-Box et al. 2017). With wild-type C57BL/6 mice as the control, we observed the ameliorative effect of Glab (40 mg/kg) on autoimmune diseases in Trex1^−/−^ mice by daily intraperitoneal injection. Tissues were collected from various parts of the mice on the fourteenth day. The histological results showed that in the heart, tongue, muscle, stomach, kidney and intestinal tissues of WT and *Trex1*^*−/−*^ mice, the WT mice showed normal histology, the *Trex1*^*−/−*^ mice showed a marked inflammatory response with increased inflammatory infiltration, and the Glab group showed a gradual reduction in the inflammatory response (Fig. 5A). This suggests that Glab exerts a therapeutic effect on the inflammatory response during this process. Meanwhile, we examined the expression of the same genes in different tissues of WT and *Trex1*^*−/−*^ mice and showed that the expression of type I interferon-related genes (IFN-β, CXCXL10 and ISG15) as well as pro-inflammatory cytokines (IL-6 and TNF-α) were consistently suppressed in the heart, tongue, muscle, stomach, kidney and intestine after 14 days of continuous administration of Glab (40 mg/kg) (Fig. 5B–G). In summary, these results suggest that Glab is effective in reducing the inflammatory response in *Trex1*^*−/−*^ mice.

## Discussion

Existing studies suggest that the cGAS-STING signaling pathway is the primary pathway that mediates DNA immune responses in vivo (Ma et al. 2015; Chen et al. 2016). cGAS is present in the cytoplasm as a pattern recognition receptor that recognizes abnormal cytoplasmic DNA, catalyzes the synthesis of cGAMP, subsequently induces SITING activation and signals to TBK1/IRF3, which ultimately induces the expression of t-type I interferon and related cytokines, and ultimately activates intrinsic immunity (Li et al. 2020). Given the role of cGAS-STING in intrinsic immunity, the search for a compound that can effectively inhibit the over-activation of this pathway is considered a promising intervention strategy for the treatment of autoimmune diseases (Decout et al. 2021; Hu et al. 2022). The results of this study show that Glab effectively inhibits the activation of the cGAS-STING signalling pathway both in vitro and in vivo. This suggests that Glab has the potential to be an effective drug candidate for autoimmune diseases mediated by the cGAS-STING signaling pathway.

Previous studies have reported that Glab exerts anti-inflammatory effects through inhibition of NF-κB and MAPK signalling pathways (Kim et al. 2010; Zhang et al. 2023). In our study, we found that Glab effectively inhibited the activation of the cGAS-STING signalling pathway caused by HT-DNA, cGAMP, DMXAA and diABZI in BMDMs, while at the genetic level, Glab also effectively inhibited the elevated expression of interferons and inflammatory factors caused by the activation of the cGAS-STING pathway. This suggests that Glab inhibits the activation of the cGAS-STING signalling pathway in vitro. Meanwhile, Glab would have no influence on poly(I:C)-induced RIG-1-MAVS pathway., indicating that Glab is a specific inhibitor of the cGAS-STING pathway in BMDMs, Interestingly, in human PBMCs, there was an inhibitory effect of Glab on multiple stimulus-induced activation of the cGAS-STING signalling pathway, and also inhibited the RIG-1-MAVs pathway to a certain extent in response to Poly(I:C) stimulation. We speculate that it may be that the molecular mechanism of action of Glab differs in different cells, and further studies may be needed to investigate the occurrence of this phenomenon.

STING plays an important role in the cGAS-STING signalling pathway. After receiving intracellular signals from the second messenger cGAMP, recruiting TBK1 to form STING-TBK1 signalosome and subsequently phosphorylating IRF3 to form dimers into the nucleus to activate related gene expression (Han et al. 2021; Ke et al. 2022). As shown in Fig. 4A, we speculate that Glab's site of action may be located downstream of STING. It was also interesting to find that transfection of wild-type IRF3 in HEK-293 could serve to activate type I interferon expression, as IRF3-5D is commonly used in such studies (Yang et al. 2022) Through the investigation of Glabridin target proteins, we found that the site of action is located on STING but does not affect STING oligomerization. Further studies revealed that Glab inhibited the interaction of STING with IRF3, but had no effect on STING-TBK1interaction. This may explain why Glab specifically inhibits the activation of the cGAS-STING signalling pathway without affecting the activation of the RIG-1-MAVS pathway.

Various causes lead to inflammation and stimulate the release of large amounts of DNA and RNA-containing cellular debris. cGAS-STING pathway is rapidly activated and initiates host immunity to rapidly defend against external adverse events, but chronic and continuous activation of this pathway is also a cause of autoimmune diseases (Ou et al. 2021; Zhang et al. 2022a, b). In the *Trex1*^*−/−*^ mouse experiments, *Trex1*^*−/−*^ mice exhibited organs that were notably diseased, as shown in Fig. 5, and the results of qPCR performed on the tissues showed elevated levels of type I interferons and inflammatory factors in all six organs. However, after two weeks of Glab administration, a significant therapeutic effect was demonstrated. These results suggest that Glab can improve autoimmune diseases mediated by the cGAS-STING pathway. However, the cGAS-STING signaling pathway is not limited to autoimmune diseases and inflammation, but plays an important role in many physiological and pathological processes, such as host defense against microbial infections, antitumor immunity, cellular senescence, and autophagy (Hou et al. 2021; Hong et al. 2023). In the present study, we found that Glabridin, a flavonoid component of licorice, could inhibit cGAS-STING signaling pathway well in vivo and in vitro, and could improve tissue inflammation well after administration to *Trex1*^*−/−*^ mice. However, whether it also has good therapeutic effects on other inflammatory conditions, or on other diseases caused by cGAS-STING, remains to be researched.

## Conclusion

In this study, we found that the active component of Glabridin in licorice flavonoids can inhibit the cGAS-STING signalling pathway both in vivo and in vitro and exert therapeutic effects in autoimmune diseases mediated by *Trex1*^*−/−*^, and also provide a reference for the use of Glabridin in the treatment of autoimmune diseases mediated by the cGAS-STING pathway.

## Data Availability

Data will be made available on request.

## References

[CR1] Bustin SA, Benes V, Garson JA, Hellemans J, Huggett J, Kubista M, Mueller R, Nolan T, Pfaffl MW, Shipley GL, Vandesompele J, Wittwer CT (2009). The MIQE guidelines: minimum information for publication of quantitative real-time PCR experiments. Clin Chem.

[CR2] Chen Q, Sun L, Chen ZJ (2016). Regulation and function of the cGAS-STING pathway of cytosolic DNA sensing. Nat Immunol.

[CR3] Chung CL, Chen JH, Huang WC, Sheu JR, Hsia CW, Jayakumar T, Hsia CH, Chiou KR, Hou SM (2022). Glabridin, a bioactive flavonoid from licorice, effectively inhibits platelet activation in humans and mice. Int J Mol Sci.

[CR4] Dai J, Zhang Y, Chen D, Chen D, Li X, Wang J, Sun Y (2021). Glabridin inhibits osteoarthritis development by protecting chondrocytes against oxidative stress, apoptosis and promoting mTOR mediated autophagy. Life Sci.

[CR5] Decout A, Katz JD, Venkatraman S, Ablasser A (2021). The cGAS-STING pathway as a therapeutic target in inflammatory diseases. Nat Rev Immunol.

[CR6] Ding C, Song Z, Shen A, Chen T, Zhang A (2020). Small molecules targeting the innate immune cGAS-STING-TBK1 signaling pathway. Acta Pharm Sin B.

[CR7] Ge N, Yan G, Sun H, Yang L, Kong L, Sun Y, Han Y, Zhao Q, Kang S, Wang X (2023) Version updates of strategies for drug discovery based on effective constituents of traditional Chinese medicine. Acupunct Herbal Med 3(3):158–179

[CR8] Graham PT, Nowak AK, Cornwall SMJ, Larma I, Nelson DJ (2022). The STING agonist, DMXAA, reduces tumor vessels and enhances mesothelioma tumor antigen presentation yet blunts cytotoxic T cell function in a murine model. Front Immunol.

[CR9] Han B, Wang X, Wu P, Jiang H, Yang Q, Li S, Li J, Zhang Z (2021). Pulmonary inflammatory and fibrogenic response induced by graphitized multi-walled carbon nanotube involved in cGAS-STING signaling pathway. J Hazard Mater.

[CR10] Hong B, Sahu U, Mullarkey MP, Hong E, Pei G, Yan Y, Otani Y, Banasavadi-Siddegowda Y, Fan H, Zhao Z, Yu J, Caligiuri MA, Kaur B (2023). PKR induces TGF-β and limits oncolytic immune therapy. J Immunother Cancer.

[CR11] Hopfner KP, Hornung V (2020). Molecular mechanisms and cellular functions of cGAS-STING signalling. Nat Rev Mol Cell Biol.

[CR12] Hou Y, Wei Y, Lautrup S, Yang B, Wang Y, Cordonnier S, Mattson MP, Croteau DL, Bohr VA (2021). NAD(+) supplementation reduces neuroinflammation and cell senescence in a transgenic mouse model of Alzheimer’s disease via cGAS-STING. Proc Natl Acad Sci USA.

[CR13] Hu Y, Chen B, Yang F, Su Y, Yang D, Yao Y, Wang S, Wu Y, Tao L, Xu T (2022). Emerging role of the cGAS-STING signaling pathway in autoimmune diseases: biologic function, mechanisms and clinical prospection. Autoimmun Rev.

[CR14] Ke X, Hu T, Jiang M (2022). cGAS-STING signaling pathway in gastrointestinal inflammatory disease and cancers. Faseb j.

[CR15] Kim JY, Kang JS, Kim HM, Ryu HS, Kim HS, Lee HK, Kim YJ, Hong JT, Kim Y, Han SB (2010). Inhibition of bone marrow-derived dendritic cell maturation by glabridin. Int Immunopharmacol.

[CR16] Kwon J, Bakhoum SF (2020). The cytosolic DNA-sensing cGAS-STING pathway in cancer. Cancer Discov.

[CR17] Li S, Hong Z, Wang Z, Li F, Mei J, Huang L, Lou X, Zhao S, Song L, Chen W, Wang Q, Liu H, Cai Y, Yu H, Xu H, Zeng G, Wang Q, Zhu J, Liu X, Tan N, Wang C (2018). The cyclopeptide astin C specifically inhibits the innate immune CDN sensor STING. Cell Rep.

[CR18] Li S, Hong Z, Wang Z, Li F, Mei J, Huang L, Lou X, Zhao S, Song L, Chen W, Wang Q, Liu H, Cai Y, Yu H, Xu H, Zeng G, Wang Q, Zhu J, Liu X, Tan N, Wang C (2018). The cyclopeptide astin C specifically inhibits the innate immune CDN sensor STING. Cell Rep.

[CR19] Li W, Lu L, Lu J, Wang X, Yang C, Jin J, Wu L, Hong X, Li F, Cao D, Yang Y, Wu M, Su B, Cheng J, Yang X, Di W, Deng L (2020). cGAS-STING-mediated DNA sensing maintains CD8(+) T cell stemness and promotes antitumor T cell therapy. Sci Transl Med.

[CR20] Li Q, Feng H, Wang H, Wang Y, Mou W, Xu G, Zhang P, Li R, Shi W, Wang Z, Fang Z, Ren L, Wang Y, Lin L, Hou X, Dai W, Li Z, Wei Z, Liu T, Wang J, Guo Y, Li P, Zhao X, Zhan X, Xiao X, Bai Z (2022). Licochalcone B specifically inhibits the NLRP3 inflammasome by disrupting NEK7-NLRP3 interaction. EMBO Rep.

[CR21] Liu L, Wang D, Liu M, Yu H, Chen Q, Wu Y, Bao R, Zhang Y, Wang T (2022) The development from hyperuricemia to gout: key mechanisms and natural products for treatment. Acupunct Herbal Med 2(1):25–32

[CR22] Lockhart A, Mucida D, Parsa R (2022). Immunity to enteric viruses. Immunity.

[CR23] Long ZJ, Wang JD, Xu JQ, Lei XX, Liu Q (2022). cGAS/STING cross-talks with cell cycle and potentiates cancer immunotherapy. Mol Ther.

[CR24] Ma Z, Jacobs SR, West JA, Stopford C, Zhang Z, Davis Z, Barber GN, Glaunsinger BA, Dittmer DP, Damania B (2015). Modulation of the cGAS-STING DNA sensing pathway by gammaherpesviruses. Proc Natl Acad Sci U S A.

[CR25] Maltbaek JH, Cambier S, Snyder JM, Stetson DB (2022). ABCC1 transporter exports the immunostimulatory cyclic dinucleotide cGAMP. Immunity.

[CR26] Mathavarajah S, Salsman J, Dellaire G (2019). An emerging role for calcium signalling in innate and autoimmunity via the cGAS-STING axis. Cytokine Growth Factor Rev.

[CR27] Nader GPF, Agüera-Gonzalez S, Routet F, Gratia M, Maurin M, Cancila V, Cadart C, Palamidessi A, Ramos RN, San Roman M, Gentili M, Yamada A, Williart A, Lodillinsky C, Lagoutte E, Villard C, Viovy JL, Tripodo C, Galon J, Scita G, Manel N, Chavrier P, Piel M (2021). Compromised nuclear envelope integrity drives TREX1-dependent DNA damage and tumor cell invasion. Cell.

[CR28] Ou L, Zhang A, Cheng Y, Chen Y (2021). The cGAS-STING pathway: a promising immunotherapy target. Front Immunol.

[CR29] Stetson DB, Ko JS, Heidmann T, Medzhitov R (2008). Trex1 prevents cell-intrinsic initiation of autoimmunity. Cell.

[CR30] Vanpouille-Box C, Alard A, Aryankalayil MJ, Sarfraz Y, Diamond JM, Schneider RJ, Inghirami G, Coleman CN, Formenti SC, Demaria S (2017). DNA exonuclease Trex1 regulates radiotherapy-induced tumour immunogenicity. Nat Commun.

[CR31] Wang Z, Xu G, Gao Y, Zhan X, Qin N, Fu S, Li R, Niu M, Wang J, Liu Y, Xiao X, Bai Z (2019). Cardamonin from a medicinal herb protects against LPS-induced septic shock by suppressing NLRP3 inflammasome. Acta Pharm Sin B.

[CR32] Wang Y, Luo J, Alu A, Han X, Wei Y, Wei X (2020). cGAS-STING pathway in cancer biotherapy. Mol Cancer.

[CR33] Wang X, Lu W, Xia X, Zhu Y, Ge C, Guo X, Zhang N, Chen H, Xu S (2022). Selenomethionine mitigate PM2.5-induced cellular senescence in the lung via attenuating inflammatory response mediated by cGAS/STING/NF-κB pathway. Ecotoxicol Environ Saf.

[CR34] Wen J, Qin S, Li Y, Zhang P, Zhan X, Fang M, Shi C, Mu W, Kan W, Zhao J, Hui S, Hou M, Li H, Xiao X, Xu G, Bai Z (2023). Flavonoids derived from licorice suppress LPS-induced acute lung injury in mice by inhibiting the cGAS-STING signaling pathway. Food Chem Toxicol.

[CR35] Yang K, Xue Y, Niu H, Shi C, Cheng M, Wang J, Zou B, Wang J, Niu T, Bao M, Yang W, Zhao D, Jiang Y, Yang G, Zeng Y, Cao X, Wang C (2022). African swine fever virus MGF360-11L negatively regulates cGAS-STING-mediated inhibition of type I interferon production. Vet Res.

[CR36] Yehuda I, Madar Z, Leikin-Frenkel A, Szuchman-Sapir A, Magzal F, Markman G, Tamir S (2016). Glabridin, an isoflavan from licorice root, upregulates paraoxonase 2 expression under hyperglycemia and protects it from oxidation. Mol Nutr Food Res.

[CR37] Zhang X, Bai XC, Chen ZJ (2020). Structures and mechanisms in the cGAS-STING innate immunity pathway. Immunity.

[CR38] Zhang D, Liu Y, Zhu Y, Zhang Q, Guan H, Liu S, Chen S, Mei C, Chen C, Liao Z, Xi Y, Ouyang S, Feng XH, Liang T, Shen L, Xu P (2022). A non-canonical cGAS-STING-PERK pathway facilitates the translational program critical for senescence and organ fibrosis. Nat Cell Biol.

[CR39] Zhang L, Zhang H, Gu J, Xu W, Yuan N, Sun J, Li H (2022). Glabridin inhibits liver fibrosis and hepatic stellate cells activation through suppression of inflammation and oxidative stress by activating PPARγ in carbon tetrachloride-treated mice. Int Immunopharmacol.

[CR40] Zhang J, Wu X, Zhong B, Liao Q, Wang X, Xie Y, He X (2023). Review on the diverse biological effects of Glabridin. Drug Des Devel Ther.

[CR41] Zhao J, Xu G, Hou X, Mu W, Yang H, Shi W, Wen J, Liu T, Wu Z, Bai J, Zhang P, Wang Z, Xiao X, Zou W, Bai Z, Zhan X (2023). Schisandrin C enhances cGAS-STING pathway activation and inhibits HBV replication. J Ethnopharmacol.

[CR42] Zhao Z, Peng Y, Shi X, Zhao K (2023). Chitosan derivative composite nanoparticles as adjuvants enhance the cellular immune response via activation of the cGAS-STING pathway. Int J Pharm.

[CR43] Zheng W, Xia N, Zhang J, Chen N, Meurens F, Liu Z, Zhu J (2021). How the innate immune DNA sensing cGAS-STING pathway is involved in autophagy. Int J Mol Sci.

[CR44] Zhou W, Whiteley AT, de Oliveira Mann CC, Morehouse BR, Nowak RP, Fischer ES, Gray NS, Mekalanos JJ, Kranzusch PJ (2018). Structure of the human cGAS-DNA complex reveals enhanced control of immune surveillance. Cell.

[CR45] Zhuang X, Ma J, Xu G, Sun Z (2022). SHP-1 knockdown suppresses mitochondrial biogenesis and aggravates mitochondria-dependent apoptosis induced by all trans retinal through the STING/AMPK pathways. Mol Med.

